# Dapagliflozin reduces albuminuria over 2 years in patients with type 2 diabetes mellitus and renal impairment

**DOI:** 10.1007/s00125-016-4017-1

**Published:** 2016-06-15

**Authors:** Paola Fioretto, Bergur V. Stefansson, Eva Johnsson, Valerie A. Cain, C. David Sjöström

**Affiliations:** 1 Clinica Medica 3, Department of Medicine, University of Padova, Via Giustiniani 2, 35128 Padova, Italy; 2 AstraZeneca, Gothenburg, Mölndal Sweden; 3 AstraZeneca, Wilmington, Delaware USA

**Keywords:** Albuminuria, Dapagliflozin, Renal impairment, SGLT2 inhibition, Type 2 diabetes

*To the Editor:* There is a growing body of evidence that sodium-glucose co-transporter 2 (SGLT2) inhibition may confer a renoprotective effect. This beneficial renal effect is thought to be achieved by mechanisms associated with reduced glucose and sodium reabsorption in the proximal tubule leading to decreased intra-glomerular pressure through the tubuloglomerular feedback mechanism [1]. In addition, reduced glucose trafficking through the proximal tubular cells [2] may lead to decreased oxidative stress, inflammation and tubulointerstitial fibrosis. Limiting proximal tubular reabsorption and, thus, reducing hyperfiltration is an important therapeutic target, since glomerular hyperfiltration is a potential driver of renal disease progression in type 2 diabetes [1]. Furthermore, changes in albuminuria predict morbidity and mortality, as well as cardiovascular and renal outcomes in patients with type 2 diabetes [3], and a short-term beneficial effect of dapagliflozin on albumin excretion has been reported [4].

The efficacy and safety of dapagliflozin in 252 patients with type 2 diabetes and moderate renal impairment has previously been assessed in a paper by Kohan and colleagues [5] . We conducted a post hoc analysis of data from this study to examine the long-term effects of dapagliflozin on urinary albumin/creatinine ratio (UACR) in patients with UACR ≥3.4 mg/mmol (≥30 mg/g) at baseline. We also examined whether changes in UACR occur independently of sex and changes in HbA_1c_, BP, uric acid and estimated GFR (eGFR).

Our post hoc analysis included 166 patients with stage 3 chronic kidney disease (CKD) and increased albuminuria (≥3.4 mg/mmol). Patients were randomised to dapagliflozin 10 mg (*n* = 56), dapagliflozin 5 mg (*n* = 53) or placebo (*n* = 57). Institutional review boards or independent ethics committees approved the protocol. Patients provided written informed consent.

Percentage change in UACR (with/without adjustments for sex and changes in HbA_1c_, systolic and diastolic BP, uric acid and eGFR), overall adverse events (AEs), AEs of special interest (AEs of renal function and volume reduction based upon a predefined list of preferred terms) and changes in eGFR, HbA_1c_, body weight and BP were assessed up to Week 104 and included data after rescue. UACR was measured at each visit of the 104-week treatment period using standard, fasting, untimed (‘spot’) morning urine samples. All samples were handled using a central laboratory procedure (Quintiles Laboratories, www.quintiles.com).

The analyses included all randomised patients with UACR ≥3.4 mg/mmol. Mean change from baseline value and 95% CI were derived using the longitudinal repeated measures mixed model with fixed terms for treatment, study week, strata (pre-enrolment anti-hyperglycaemic therapy was defined as: insulin [INS] ± another anti-hyperglycaemic medication or sulfonylurea [SU] ± anti-hyperglycaemic except INS or thiazolidinedione-based regimen except SU or INS or any anti-hyperglycaemic agent[s] not previously described or no background anti-hyperglycaemic medication) study week-by-treatment interaction as well as the fixed covariates of baseline and baseline-by-week interaction. The model also included an indicator variable to indicate if rescue had occurred at each visit. UACR values were log transformed (using the natural log) and then exponentiated back to the original scale. The shift in albuminuria status was assessed from baseline to Week 104.

Adverse event data were summarised using descriptive statistics. All analyses for both safety and efficacy variables also included data from patients who had received glycaemic rescue therapy. Patients received open-label rescue therapy with an anti-hyperglycaemic agent (except metformin) if pre-defined rescue criteria were exceeded. Changes in antihypertensive medications were not controlled for in this study. Baseline characteristics were largely comparable across groups (electronic supplementary material [ESM] Table 1). Median (range) UACR was 20.2 (3.6–541.5), 44.9 (3.5–561.6) and 20.3 (3.4–1046.6) mg/mmol in the dapagliflozin 10 mg, 5 mg and placebo groups, respectively.

Placebo-corrected UACR reductions (95% CI) of −57.2% (−77.1, −20.1) and −43.8% (−71.0, 9.0) occurred in the dapagliflozin 10 mg and 5 mg groups, respectively, at 104 weeks (Fig. 1a). UACR measurements were available for 29, 20 and 25 patients in the dapagliflozin 10 mg, 5 mg and placebo groups, respectively, at 104 weeks. After adjusting for sex and changes in BP, HbA_1c_, eGFR and uric acid, placebo-corrected reductions (95% CI) of −53.6% (−75.5, −12.1) and −47.4% (−73.7, 5.3) were observed in the dapagliflozin 10 mg and 5 mg, respectively (ESM Fig. 1), indicating that the renal effects of dapagliflozin were largely independent of changes in these variables.

**Fig. 1:**
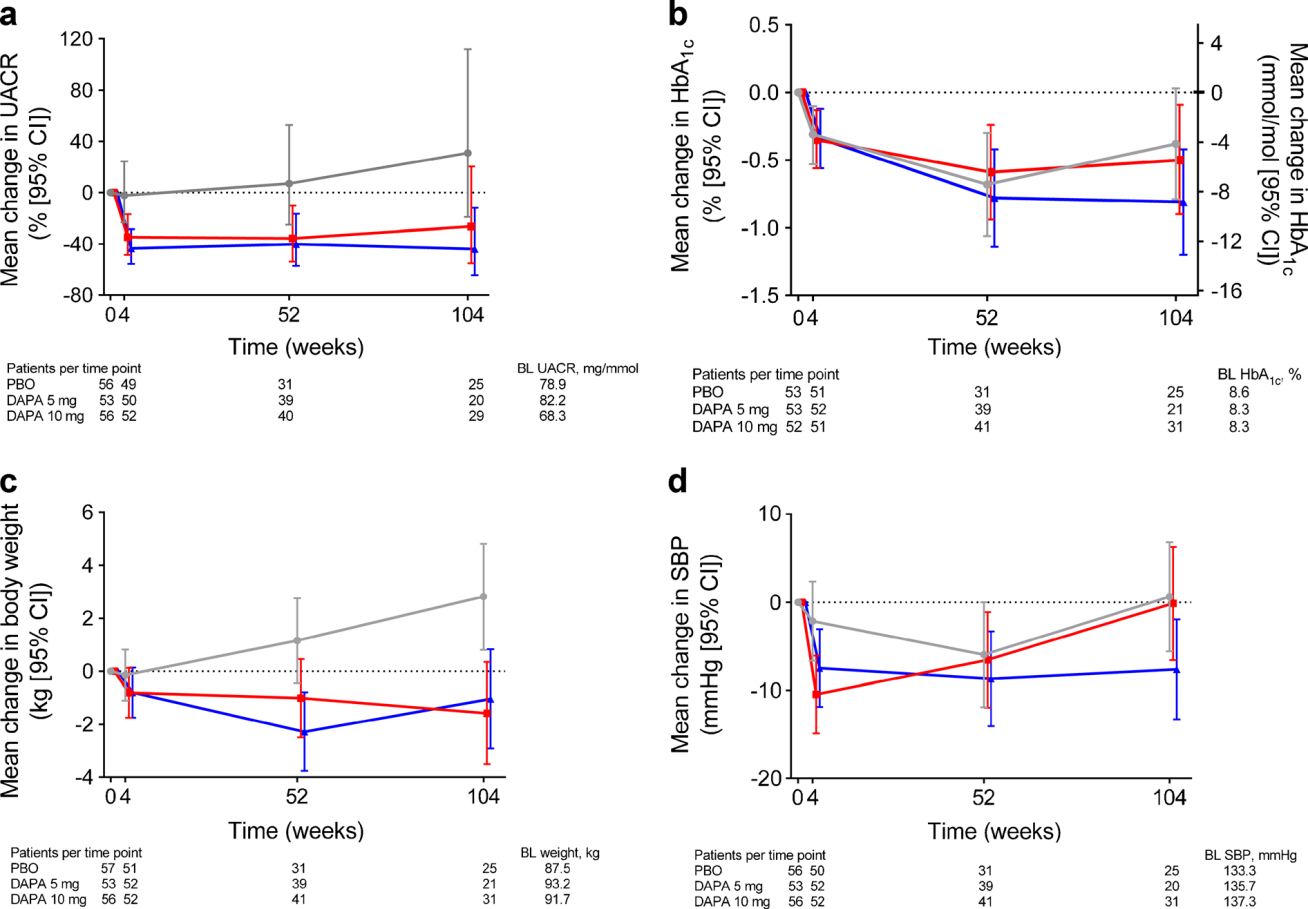
Adjusted mean changes (95% CI) in (**a**) UACR, (**b**) HbA_1c_, (**c**) body weight and (**d**) systolic BP, for dapagliflozin (DAPA) 10 mg, DAPA 5 mg and PBO, over 104 weeks. Mean change from baseline data (95% CI) were derived using the longitudinal repeated measures mixed model with fixed terms for treatment, study week, strata (pre-enrolment anti-hyperglycaemic therapy) study week-by-treatment interaction, as well as the fixed covariates of baseline and baseline-by-week interaction. The model also included an indicator variable to indicate if rescue had occurred at each visit. (**a**) Adjusted mean change in UACR at Week 104 for DAPA 10 mg: −43.9 (−64.3, −12.0); DAPA 5 mg: −26.4 (−55.0, 20.5) and PBO: 31.0 (−19.0, 111.9). (**b**) Adjusted mean change in HbA_1c_ at Week 104 for DAPA 10 mg: −0.8 (−1.2, −0.4); DAPA 5 mg: −0.5 (−0.9, −0.1) and PBO: −0.4 (−0.8, 0.0). (**c**) Adjusted mean change in body weight at Week 104 for DAPA 10 mg: −1.6 (−3.5, 0.4); DAPA 5 mg: −1.0 (−2.9, 0.8) and PBO: 2.8 (0.8, 4.8). (**d**) Adjusted mean change in systolic BP at Week 104 for DAPA 10 mg: −7.6 (−13.3, −1.9); DAPA 5 mg: 0.1 (−6.6, 6.3) and PBO: 0.6 (−5.6, 6.9). Blue triangles, dapagliflozin 10 mg; red squares, dapagliflozin 5 mg; grey circles, placebo. BL, baseline; DAPA, dapagliflozin; PBO, placebo; SBP, systolic blood pressure

Compared with placebo, more patients in the dapagliflozin 10 mg and 5 mg groups shifted to a lower UACR category (33.9 and 39.6%, respectively, vs 15.8% with placebo) and fewer progressed to a higher UACR category (14.7% and 4.3% respectively, vs 27.3% with placebo) (ESM Fig. 2). Overall, 17.8%, 18.9% and 7.0% of patients improved to normoalbuminuria status in the dapagliflozin 10 mg, 5 mg and placebo groups, respectively.

There was an initial decrease in eGFR within the first 4 weeks of dapagliflozin therapy with no further decline over the 104 weeks, whereas the placebo-treated patients showed a gradual decline over the entire study period (ESM Fig. 3).

Dapagliflozin 10 mg and 5 mg groups showed placebo-corrected HbA_1c_ reductions (95% CI) of −0.43% (−0.95, 0.10) (−4.7 mmol/mol [−10.4, 1.1]) and −0.11% (−0.65, 0.42) (−1.2 mmol/mol [−7.1, 4.6]), respectively, at 104 weeks (Fig. 1b). Dapagliflozin 10 mg and 5 mg groups also showed placebo-corrected reductions (95% CI) of −3.9 kg (−6.4, −1.3) and −4.4 kg (−7.0, −1.8) in weight (Fig. 1c). Placebo-corrected reductions (95% CI) in systolic BP were numerically greater with dapagliflozin 10 mg (−8.3 mmHg [−16.2, −0.3]) vs dapagliflozin 5 mg (−0.8 mmHg [−9.2, 7.7]) (Fig. 1d). Placebo-corrected reductions (95% CI) in uric acid were −12.5 (−47.0, 22.0) and −35.1 (−70.8, 0.6) μmol/l in the dapagliflozin 10 mg and 5 mg groups, respectively (data not shown).

Renal AEs were more common in the dapagliflozin 10 mg treated patients (10.7%) vs those on dapagliflozin 5 mg (1.9%) or placebo (3.5%); these events were mostly associated with increased creatinine (ESM Table 2). There was no increase in serious AEs of renal function in the dapagliflozin 10 mg and 5 mg groups (1.8% and 1.9%, respectively) vs placebo (1.8%) (ESM Table 2). AEs of volume reduction were balanced across groups (8.9%, 9.4% and 7.0% in the dapagliflozin 10 mg, 5 mg and placebo groups, respectively). One serious AE of volume reduction (syncope) was reported in the dapagliflozin 10 mg group. The most common AEs leading to discontinuation were related to hyperkalaemia, with a greater frequency noted with placebo vs dapagliflozin (ESM Table 2).

A limitation of this analysis is that it is a post hoc analysis with a relatively small sample size. Nevertheless, reductions in albuminuria, along with an indication of a long-term delay in worsening eGFR suggest that dapagliflozin may have a favourable effect on preventing/delaying progression of renal disease. Moreover, recently published data have shown dapagliflozin-induced reductions in albuminuria at 12 weeks in patients receiving renin-angiotensin system blockade therapy [4]. This hypothesis is further supported by a recent empagliflozin trial, that showed significant improvements in hard renal outcomes in patients with type 2 diabetes, cardiovascular disease and various degrees of CKD [6].

In conclusion, dapagliflozin reduced UACR over two years in individuals with type 2 diabetes and stage 3 CKD, without increases in serious renal AEs. The efficacy and safety of dapagliflozin in individuals with type 2 diabetes, albuminuria and moderate renal impairment is being further evaluated in an ongoing study (NCT02547935). Other, long-term trials of SGLT2 inhibitors exploring renal endpoints (NCT01989754, NCT02065791, NCT01730534) are underway to help to further characterise their potential renal benefits in type 2 diabetes.

*Trial registration*: ClinicalTrials.gov NCT00663260

*Funding*: This study was funded by AstraZeneca

## Electronic supplementary material

Below is the link to the electronic supplementary material.

ESM (PDF 165 kb)

## Abbreviations

AE: Adverse event
CKD: Chronic kidney disease
eGFR: Estimated glomerular filtration rate
SGLT2: Sodium-glucose co-transporter 2
UACR: Urinary albumin/creatinine ratio
