# Supplementary material for: Dapagliflozin reduces albuminuria over 2 years in patients with type 2 diabetes mellitus and renal impairment
Source: Diabetologia. 2016 Jun 15;59:2036–9. doi: 10.1007/s00125-016-4017-1 (PMC4969341; doi:10.1007/s00125-016-4017-1)
Supplement: Supplementary file 1 — (PDF 165 kb) [file 125_2016_4017_MOESM1_ESM.pdf]

Electronic supplementary material

ESM Table 1: Demographics and baseline characteristics

	DAPA 10 mg (n=56)	DAPA 5 mg (n=53)	Placebo (n=57)
**Age, years**	68 (8.4)	65 (9.8)	66 (8.3)
**Age categorization, n (%)**			
<65 years	19 (33.9)	24 (45.3)	27 (47.4)
≥65 and <75 years	25 (44.6)	24 (45.3)	19 (33.3)
≥75 years	12 (21.4)	5 (9.4)	11 (19.3)
**Race, n (%)**			
White	50 (89.3)	41 (77.4)	46 (80.7)
Asian	2 (3.6)	1 (1.9)	5 (8.8)
Black/African-American	3 (5.4)	5 (9.4)	0 (0)
Other	1 (1.8)	6 (11.3)	6 (10.5)
**Male, n (%)**	44 (78.6)	36 (67.9)	36 (63.2)
**HbA_{1c}**			
%	8.3 (1.0)	8.3 (1.0)	8.6 (1.3)
mmol/mol	67 (11.0)	67 (11.0)	70 (14.1)
**Body mass index, kg/m^2**	32.4 (5.7)	33.0 (5.4)	32.2 (6.4)
**Systolic blood pressure, mmHg**	137.3 (17.7)	135.7 (18.5)	133.3 (14.1)[a]
**Diastolic blood pressure, mmHg**	75.1 (9.7)	74.3 (9.3)	74.6 (10.1)[a]
**Duration of T2DM, years**	18.2 (10.1)	18.5 (8.5)	17.0 (9.6)
**UACR, mg/mmol, median (range)**	20.2 (3.6–541.5)	44.9 (3.5–561.6)	20.3 (3.4–1046.6)[a]
**Proportion of patients with macro albuminuria, n (%)**	22 (39.3)	30 (56.6)	24 (42.1)
**eGFR, ml/min/1.73 m^2**	44.1 (11.1)	43.9 (9.0)	45.1 (9.4)[a]
**Uric acid, µmol/l**	423 (95)	425 (118)	410 (117)[a]
**Pre-enrolment anti-hyperglycaemic therapy, n (%)**			
Insulin-based regimen	35 (62.5)	36 (67.9)	38 (66.7)
SU-based regimen	16 (28.6)	11 (20.8)	15 (26.3)
TZD-based regimen	1 (1.8)	1 (1.9)	1 (1.8)
Other regimen	4 (7.1)	5 (9.4)	3 (5.3)
**Pre-enrolment CV therapy, n (%)**			
ACEi/ARB therapy	46 (82.1)	45 (84.9)	51 (89.5)
Diuretics, overall	36 (64.3)	34 (64.2)	29 (50.9)
Loop diuretics	16 (28.6)	20 (37.7)	18 (31.6)
**Pre-enrolment medical history, n (%)**			
Diabetic retinopathy	21 (37.5)	26 (49.1)	21 (36.8)
Hypertension	54 (96.4)	50 (94.3)	51 (89.5)
Coronary artery disease	18 (32.1)	18 (34.0)	20 (35.1)

Data are mean (SD) unless otherwise stated. [a]Placebo group: n=56 for baseline UACR, eGFR, systolic, uric acid and diastolic blood pressure. ACEi, angiotensin converting-enzyme inhibitor; ARB, angiotensin receptor blocker; CV, cardiovascular; DAPA, dapagliflozin; eGFR, estimated glomerular filtration rate; SU, sulfonylurea; T2DM, type 2 diabetes mellitus; TZD, thiazolidinedione; UACR, urinary albumin:creatinine ratio.

ESM Table 2: Adverse events over 104 weeks

	DAPA 10mg (n=56)	DAPA 5 mg (n=53)	Placebo (n=57)
**Overall AEs and SAEs**			
**≥1 AE[a]**	50 (89.3)	50 (94.3)	53 (93.0)
**AE leading to discontinuation[a,b]**	8 (14.3)	12 (22.6)	19 (33.3)
**≥1 hypoglycaemia[c]**	22 (39.3)	24 (45.3)	31 (54.4)
**Hypoglycaemia leading to discontinuation[c]**	0 (0)	0 (0)	1 (1.8)
**≥1 SAE[a]**	20 (35.7)	14 (26.4)	18 (31.6)
**SAE leading to discontinuation**	2 (3.6)	5 (9.4)	5 (8.8)
**Death**	3 (5.4)	1 (1.9)	4 (7.0)
**AEs and SAEs of renal function[d]**			
**AEs of renal function**	6 (10.7)	1 (1.9)	2 (3.5)
**Blood creatinine increased**	3 (5.4)	0 (0)	1 (1.8)
**Renal failure**	0 (0)	1 (1.9)	1 (1.8)
**Renal failure chronic**	1 (1.8)	0 (0)	0 (0)
**Renal impairment**	2 (3.6)	0 (0)	0 (0)
**SAEs of renal function**	1 (1.8)	1 (1.9)	1 (1.8)
**Renal failure**	0 (0)	1 (1.9)	1 (1.8)
**Renal failure chronic**	1 (1.8)	0 (0)	0 (0)
**AEs and SAEs of volume reduction[d]**			
**AEs of volume reduction**	5 (8.9)	5 (9.4)	4 (7.0)
**Blood pressure decreased**	0 (0)	1 (1.9)	1 (1.8)
**Dehydration**	1 (1.8)	0 (0)	1 (1.8)
**Syncope**	1 (1.8)	0 (0)	0 (0)
**Hypotension**	3 (5.4)	2 (3.8)	1 (1.8)
**Orthostatic hypotension**	1 (1.8)	2 (3.8)	2 (3.5)
**SAEs of volume reduction**	1 (1.8)	0 (0)	0 (0)
**Syncope**	1 (1.8)	0 (0)	0 (0)

[a]Only hypoglycaemia reported as a SAE is included in the AE, SAE and AE leading to discontinuation summary lines. [b]The most common AEs that led to discontinuation were related to the preferred terms of hyperkalaemia (4, 4 and 8 events registered in the dapagliflozin 10mg, dapagliflozin 5mg and placebo groups, respectively) and increased blood potassium (0, 1 and 6 events registered, respectively). [c]All reported hypoglycaemia events are included in the hypoglycaemia line. [d]Based on a predefined list of events of renal impairment /volume reduction. AE, adverse event; DAPA, dapagliflozin; SAE, serious adverse event.

ESM Fig. 1: Placebo-corrected per cent change in albuminuria with/without adjustment for sex and changes in HbA_{1c}, systolic and diastolic blood pressure, uric acid and eGFR at 104 weeks

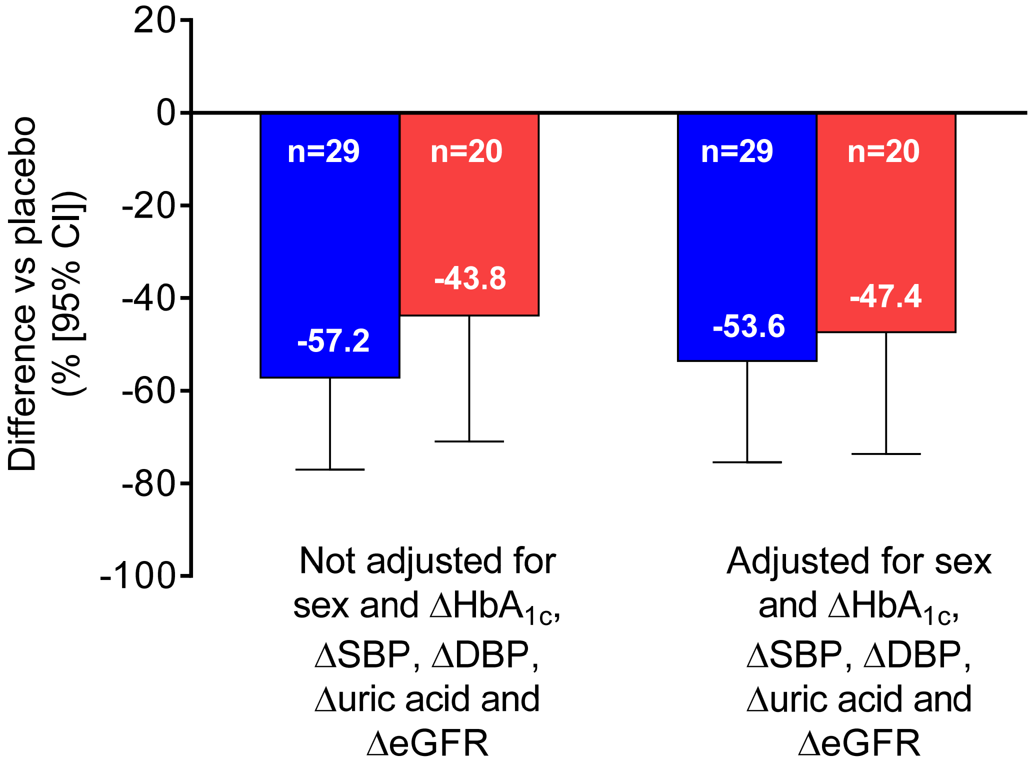

DBP, diastolic blood pressure; eGFR, estimated glomerular filtration rate; SBP, systolic blood pressure.

Blue bars represent dapagliflozin 10mg and red bars represent dapagliflozin 5mg.

ESM Fig. 2: Patients (%) shifting UACR level from baseline to 104 weeks

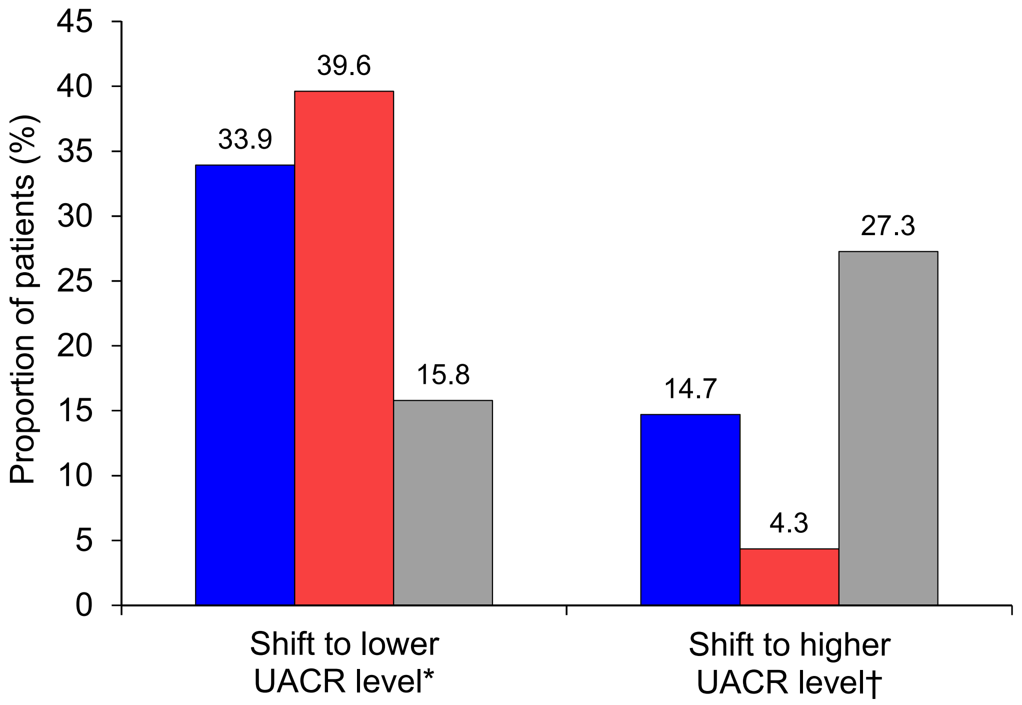

UACR, urinary albumin:creatinine ratio. Placebo: n=57; Dapagliflozin 5 mg: n=53; Dapagliflozin 10 mg: n=56. *Patients shifting from micro- to normoalbuminuria or from macro- to micro- or normoalbuminuria. †Patients shifting from micro- to macroalbuminuria.

Blue bars represent dapagliflozin 10mg, red bars represent dapagliflozin 5mg and grey bars represent placebo.

ESM Fig. 3: Mean change in eGFR over 104 weeks

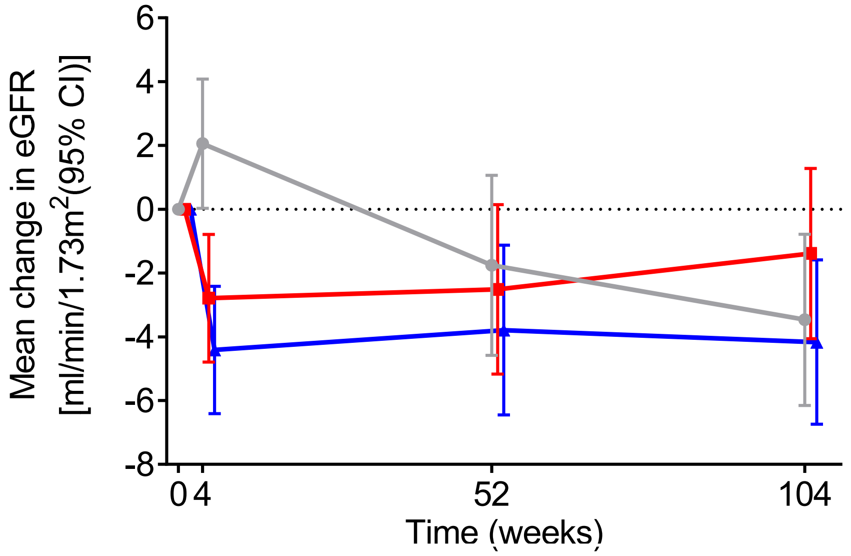

Patients per time point					BL eGFR, mL/min/1.73m^2
PBO	56	50	31	25	45.1
DAPA 5 mg	53	52	39	20	43.9
DAPA 10 mg	56	52	41	30	44.1

Adjusted mean change in eGFR at Week 104 for DAPA 10 mg: −4.2 (−6.7, −1.6); DAPA 5 mg: −1.4 (−4.1, 1.3) and PBO: −3.5 (−6.2, −0.8)

Difference vs placebo (95% CI) in eGFR at Week 104 for DAPA 10mg: −0.7 ml/min/1.73 m^2 (−4.0, 2.6); DAPA 5 mg: 2.1 ml/min/1.73 m^2 (−1.3, 5.5). DAPA, dapagliflozin; eGFR, estimated glomerular filtration rate; PBO, placebo.

Blue triangles represent data points for dapagliflozin 10mg, red squares for dapagliflozin 5mg and grey circles for placebo.
